# Improved Antibiotic Detection in Raw Milk Using Machine Learning Tools over the Absorption Spectra of a Problem-Specific Nanobiosensor

**DOI:** 10.3390/s20164552

**Published:** 2020-08-14

**Authors:** Pablo Gutiérrez, Sebastián E. Godoy, Sergio Torres, Patricio Oyarzún, Ignacio Sanhueza, Victor Díaz-García, Braulio Contreras-Trigo, Pablo Coelho

**Affiliations:** 1 Departamento de Ingeniería Eléctrica, Facultad de Ingeniería, Universidad de Concepción, Concepción 4030000, Chile; pablogutierrez@udec.cl (P.G.); segodoy@udec.cl (S.E.G.); sertorre@udec.cl (S.T.); ignacio.sanhueza.ruiz@gmail.com (I.S.); 2 Facultad de Ingeniería y Tecnología, Universidad San Sebastián, Lientur 1457, Concepción 4080871, Chile; victor.diazg@uss.cl (V.D.-G.); bcontrerast@docente.uss.cl (B.C.-T.); pablo.coelho@uss.cl (P.C.)

**Keywords:** antibiotics, raw milk, gold nanoparticles, machine learning, nanotechnology, biosensors, absorption spectra

## Abstract

In this article we present the development of a biosensor system that integrates nanotechnology, optomechanics and a spectral detection algorithm for sensitive quantification of antibiotic residues in raw milk of cow. Firstly, nanobiosensors were designed and synthesized by chemically bonding gold nanoparticles (AuNPs) with aptamer bioreceptors highly selective for four widely used antibiotics in the field of veterinary medicine, namely, Kanamycin, Ampicillin, Oxytetracycline and Sulfadimethoxine. When molecules of the antibiotics are present in the milk sample, the interaction with the aptamers induces random AuNP aggregation. This phenomenon modifies the initial absorption spectrum of the milk sample without antibiotics, producing spectral features that indicate both the presence of antibiotics and, to some extent, its concentration. Secondly, we designed and constructed an electro-opto-mechanic device that performs automatic high-resolution spectral data acquisition in a wavelength range of 400 to 800 nm. Thirdly, the acquired spectra were processed by a machine-learning algorithm that is embedded into the acquisition hardware to determine the presence and concentration ranges of the antibiotics. Our approach outperformed state-of-the-art standardized techniques (based on the 520/620 nm ratio) for antibiotic detection, both in speed and in sensitivity.

## 1. Introduction

The growth of the world population has required the food industry to increase its productivity and improve food safety. Milk consumption either before or after thermal processing has several health risks associated with the presence of contaminants such as thermoresistant mycotoxins and antibiotic residues. Antibiotics are primarily used in dairy farms for the treatment of mastitis and other infectious diseases [1]. The World Health Organization (WHO) notes that there is ample scientific evidence that food-production animals (milk, meat and eggs) are a reservoir of antimicrobial resistant bacteria [2]. Multiresistance can reach the human population through the food chain, due to a continuous flow of resistance genes that could transfer to the human gut microbiota [3].

In Chile, the Servicio Agrícola y Ganadero (SAG) and the Ministerio de Salud (MINSAL) are the institutions responsible for ensuring the sanitary inspection and control of veterinary pharmaceutical products. The MINSAL implements a national plan to control the presence of antibiotic residues in dairy products, referred to as “Surveillance program for residues of veterinary medicines”. This program supervises the Maximum Residual Limits (MRLs) of veterinary drugs potentially present in Chilean products [4]. Milk contaminated with residues of antibiotics at levels above the MRLs must be discarded before being industrialized, preventing these contaminants from entering the human food chain and their corresponding impact on food safety and public health. In view of this, it is paramount to improve the monitoring and control of antibiotic residues in milk and dairy products, thus preventing spread of antibiotic resistance emergence and spread of antibiotic resistance [5,6].

Currently, “screening kits” are used in dairy plants for rapid antibiotic residue testing in milk, which include Delvotest${}^{®}$ [7] (standard microbiological method) and other bioanalytical methods based on binding to selective protein receptors (e.g., SNAP${}^{®}$ beta-lactam tests), which provide results in short times and with high specificity. However, these methods are semiquantitative and not developed for simultaneous detection of multiple groups of antibiotics (multidetection). Finally, the category of instrumental methods are those with the highest analytical performance, being recognized as the official confirmation technique for screening tests. Confirmatory methods are based on high-performance liquid chromatography (HPLC) coupled to mass spectrometry, which is recommended due to its capacity to perform separation, identification and quantitative analysis of analytes with high sensitivity and selectivity at very low concentrations. However, expensive and laborious instrumental methods are required to carry out the HPLC analysis.

The development of biosensing systems has opened a new and promising way to use fast, sensitive and simple techniques for the detection of pollutants in the environmental and agri-food area [8]. Nanotechnology-based biosensors (nanobiosensors; NBS) are characterized by their high specificity and sensitivity that allows the detection of a broad spectrum of analytes in complex samples. A growing number of reports has described the succesful development of these systems for antibiotic detection using aptamers as bioreceptors [9,10,11,12]. Aptamers are short single-stranded oligonucleotides with three-dimensional structure delivering high-affinity and high-specificity recognition for non-nucleotide analytes [13].

Optical NBS based on Surface Plasmon Resonance (SPR) are one of the most promising label-free sensor systems [14,15]. The SPR is an inherent optical property of noble metal nanoparticles, such as gold and silver, which results from collective oscillations of the electron clouds and allows to implement optical detection in visible range by following the shift of the absorption/emission spectrum. This property is dependent on the dimensions and geometry of the nanoparticles. Therefore, it becomes possible to design optical NBS whose interaction with a given analyte causes the nanoparticles to aggregate and, consequently, to absorb or scatter light in different spectral regions that can be instrumentally detected and quantified [16].

Novel NBS based on gold nanoparticles (AuNPs) for antibiotic detection in milk have been previously reported [9,17,18]. A successful multidetection in milk of the antibiotics Sulfadimethoxine, Kanamycin and adenosine was also reported using an AuNP-aptamer biosensor with visual colorimetric detection to determine the antibiotics at concentrations of 100–500 ng/mL [19]. In general, state-of-the-art research on biosensors for antibiotic detection calculate the concentration based on simple linear regressions of calibration curves. However, the processing and analysis of spectral signals using advanced mathematical methods, such as classification algorithms, are powerful tools that can be implemented to improve detection and analytical parameters of optical NBS [20,21].

In this article, the development of a technological solution to the problem of detecting antibiotic residues in raw milk is presented.

Current methods for the detection and/or determination of antibiotics in milk are divided into two major categories. The first group consists of screening methods, which are economical and easy to use. However, these methods are qualitative (e.g., microbiological tests) or semi-quantitative (e.g., immunoassays) and are characterized by having low sensitivity and low specificity [22]. Charm${}^{®}$ and Delvotest${}^{®}$ kits are among the most extensively employed by the dairy industry for fast (8–30 min) and sensitive milk residue screening, which allow for semi-quantitative detection of several antibody families [23]. The second group of confirmatory methods is mainly based on high-performance liquid chromatography (HPLC), which are required for quantification and validation of screening tests thanks to their excellent sensitivity and specificity [24]. These methods are not suitable for routine milk analysis, due to complex and expensive equipment requirements, and the need for qualified/experienced personal in chromatographic techniques. In this context, we support that a combination of portability and high-analytical performance would be optimal to implement monitoring programs within farms or dairies for detection and sensitive quantification of antibiotic residues in raw milk (on-farm detection). In this article, we address the development of a technological solution to the problem of optical detection of antibiotic residues present in raw milk by an SPR-based NBS, which holds promise for on-farm detection by improving analytical performance as compared with antibiotic screening kits.

The proposed device integrates optomechanical and optoelectronic components (light source, optical fiber and radiometer) to measure the variation in the absorption spectrum of the milk that is induced by the interaction between the NBS and the antibiotic molecules. Importantly, the device implements a machine-learning algorithm to simultaneously analyze optical signals from multiple samples (in a microwell plate) and determine the corresponding antibiotic concentrations.

The paper is organized as follows. The methodology used to synthesize the NBS and the analytical conditions (antibiotics, NBS, milk and preanalytical processes) are shown in the beginning of Section 2. The system software (calibration, control and classification algorithm) and hardware (electro-opto-mechanic) are shown by the end of Section 2, while the most important results and their comparison with the standardized 520/620 ratio technique are shown in Section 3. Finally, in Section 4 the main conclusions are summarized.

## 2. Material and Methods

This section depicts important aspects of the methodologies that are implemented in this work to perform the detection of antibiotics in raw cow milk.

### 2.1. Nanobiosensors and Antibiotics

**Figure 1:**
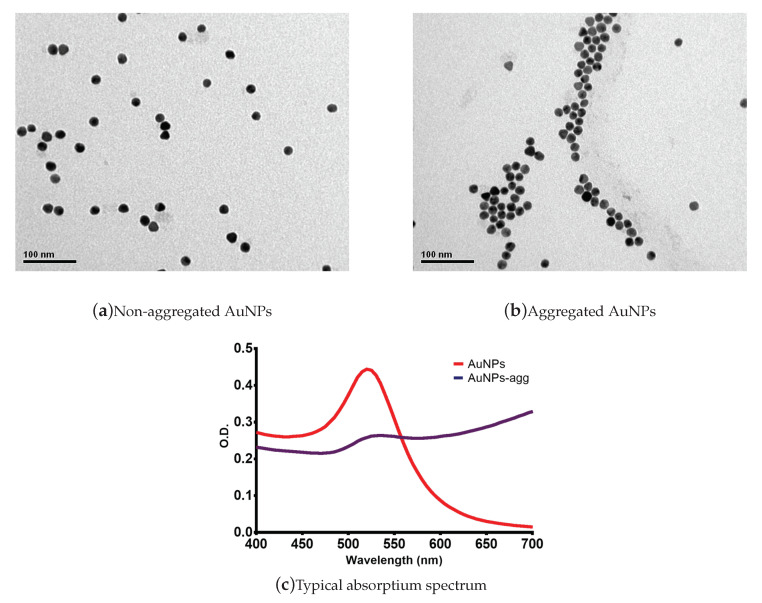
Aggregation of gold nanoparticles (AuNPs) and the corresponding absorption spectrum. (**a**) Microscopic images of non-aggregated AuNPs. (**b**) Microscopic images of aggregated AuNPs. These images where obtained using transmission electron microscopy for visual and morphological characterization (JEOL JEM 1200EX II Microscope, Electron Microscopy Laboratory, Universidad de Concepción). (**c**) Absorption spectrum of aggregated and non aggregated AuNPs. When the aggregation occurs, it produces a shift in the intensity peak of the absorption spectrum with the corresponding colorimetric change.

NBS employed in this study are devises using gold nanoparticles (AuNPs) and aptamers that specifically recognize (detect) the selected antibiotics and allow the selective aggregation of the nanoparticles, which otherwise would be a spontaneous phenomenon in aqueous solution. AuNPs were synthesized using the citrate reduction method, maintaining the synthesis pH between 5.3 and 6.0 [25]. Herein, the AuNPs were functionalized with 3${}^{\prime }$ thiol-DNA aptamers [26], according to experimental conditions previously optimized in our laboratory. The following molar ratios (AuNPs: aptamers) for each selected antibiotic were employed for NBS synthesis: (i) NBS for Kanamycin, 1:60; (ii) NBS for Ampicillin, 1:20; (iii) NBS for Oxytetracycline, 1:20 and (iv) NBS for Sulfadimethoxine, 1:20. Figure 1 displays images obtained with transmission electron microscopy of synthesized AuNPs, including non-aggregated (Figure 1a) and aggregated (Figure 1b). The corresponding absorption spectra are shown in Figure 1c, highlighting the typical peak shift associated to AuNP aggregation (purple line).

### 2.2. Milk, a Complex Matrix

This is a heterogeneous fluid held in multi-dispersed phases of emulsion (fat–water), colloidal suspension (protein–water) and solution (salts–water). Samples were prepared before applying any NBS analysis, using raw milk that was kindly facilitated by the company Leches del Bio-Bio S.A. Thus, the antibiotics Kanamycin, Ampicillin, Oxytetracycline and Sulfadimethoxine were first added into the samples at concentrations of 0.25, 0.5, 1, 1.5 and 2 times the MRL, according to the Chilean regulation in 2019 [27]. We also kept control samples (0 times MRL or 0ppb). Upon addition of the antibiotics, the milk was homogenized for 30 min at room temperature (15–25 ${}^{\circ }$C) [12,28]. As a result that optical density of raw samples was too high as to allow detection, a clarified milk was obtained by removing lipids and proteins from the fluid using a commercial clarification reagent kit for sample preparation in food analysis (Carrez method) [29], according to the manufacturer’s instructions (Merck KGaA, Darmstadt, Germany). Residuals cations were additionally removed by treating the clarified milk (1.5 mL) with 80 μL of HCO_3_ 0.6 M and incubated at 60 ${}^{\circ }$C for 10 min. The solutions were cooled at room temperature, then centrifuged at 20,000× *g* for 10 min and the resulting supernatant was recovered for the NBS experiments. In order to generate absorption spectral databases, tests were carried out in 96-well plates of clarified milk with the aforementioned concentrations for the four selected antibiotics. Finally, the NBSs were added into the clarified milk and NaCl was incorporated to trigger aggregation of the AuNPs (revealing agent).

### 2.3. Buit-In Instrument to Spectral Acquisition and Processing

In this section we describe a built-in electro-opto-mechanic prototype that acquires the spectra of the individual samples in the microplate, processes the data and correlates the measured spectrum with the concentration of the measured antibiotic.

#### Hardware and Software

**Figure 2:**
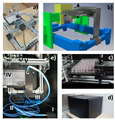
Development stages of the proposed device. (**a**) First laboratory prototype; (**b**) industrial design to address the drawbacks of the laboratory prototype; (**c**) microreactors and optical colimators; (**d**) prototype container and (**e**) key internal components: (**I**) an Ocean Optics STS VIS-NIR spectrometer, (**II**) P600 optical fibers that transmit from ultraviolet to the visible range spectrum, (**III**) an Ocean Optics HL-2000-HP-FHSA light source, (**IV**) 96-well plates for placing the samples to be measured and (**V**) a two-dimensional high-precision displacement unit to move the microplate and place the sample to be measured under the radiometer.

The hardware of the prototype is shown in Figure 2. The components of the prototype are a Ocean Optics STS VIS-NIR Spectrometer, two P600 optical fibers -025-UV–VIS from Ocean Optics, a high-power tungsten halogen light source (360–2400 nm) Ocean Optics HL-2000-HP-FHSA, a 96-well plate for placing the samples to be measured with its custom-made support, and a high-precision electro-mechanic stage controlled by software. The laboratory prototype is shown in Figure 2a, which includes several moving elements that had to be adjusted to align the optical components. Figure 2b–e present the design and industrial manufacturing of the parts to increase the robustness of the device for on-site applications.

The data acquisition and control software were developed in LabView, which allowed to define the wells to be measured and the position of the reference sample. To start the measurements, the optomechanic system moves the 96-well microplate to correctly align the reference well with the instruments, then turning on the reference well and user-defined wells are covered. The light source is subsequently turned on and the resulting transmission spectrum for each sample are acquired.

**Figure 3:**
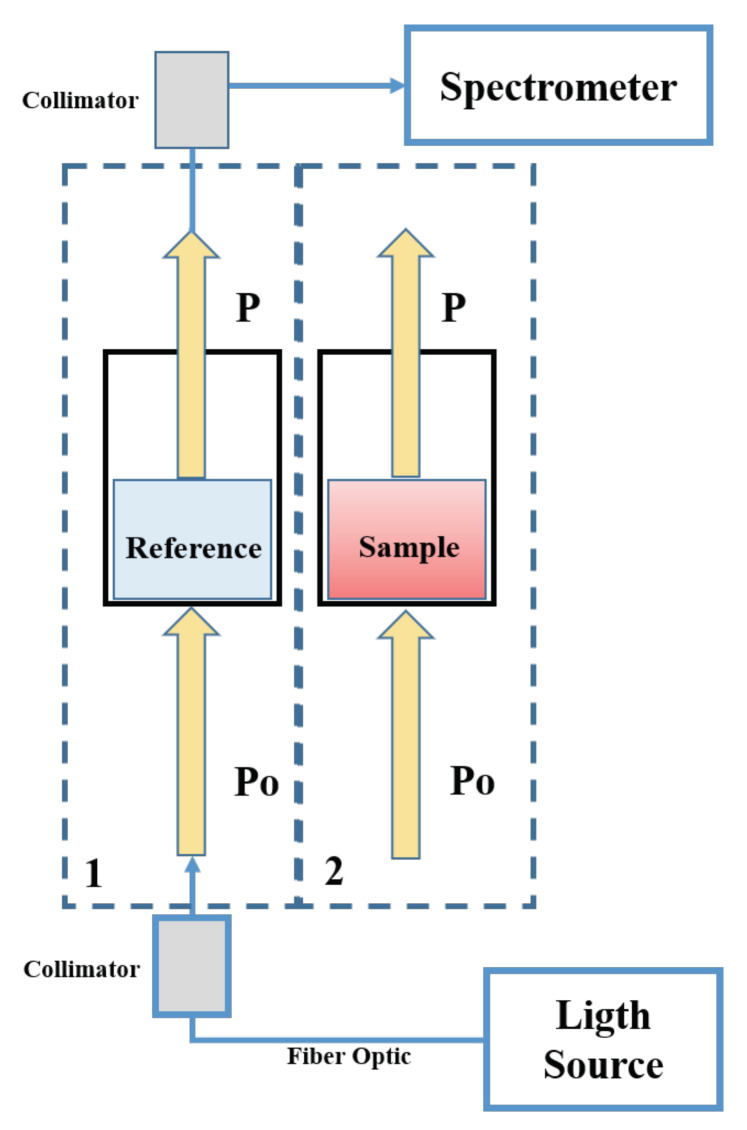
Block diagram of the optical data acquisition system. Firstly, a reference well is measured to estimate the aggregated spectrum of the container, the water and the dissolved NBS. The reference data are stored by the developed software. Secondly, each sample is measured in the same way and the NBS absorbance is obtained by subtracting the reference from the sample absorbance.

We denoted the incident radiation generated by the light source as $P_{0}\left(\lambda \right)$, and the maximum transmitted radiation of an individual well as P(λ), the transmitted radiation (transmittance) of one well is defined by $T_{total}\left(\lambda \right)=\frac{P(\lambda )}{P_{0}\left(\lambda \right)}$. The total absorbance is computed as the natural logarithm of the transmittance, namely $A_{total}\left(\lambda \right)=ln\frac{P_{0}\left(\lambda \right)}{P(\lambda )}$. The total absorbance is composed by the absorbance of the plastic microplate, $A_{mp}\left(\lambda \right)$, the absorbance of the water containing the NBS, $A_{wp}\left(\lambda \right)$ and the absorbance of the NBS, $A_{nb}\left(\lambda \right)$. As such, to determine the absorbance of interest, i.e., the absorbance of the NBS, we require a reference measurement of the pure functionalized NPs dissolved in the same purified water that is used in the contaminated milk. Thus, the reference absorbance measured from one of the wells, $A_{ref}=A_{mp}+A_{wp}$ can be simply subtracted from the total absorbance $A_{total}$ to obtain the absorbance generated by the plasmonic effect of the NBS. Figure 3 depicts a schematic of the procedure.

To reduce the effect of spectral variations of the the samples caused by small optical misalignment, sample bubbles and vibrational disturbances, the acquisition system performs multiple absorbance measurements for each well and averages the spectra before reporting the NBS spectrum.

### 2.4. Antibiotic Detection Algorithm

At this stage, for each of the four antibiotics we obtained multiple measurements for each fraction of the MRL concentrations, as shown later. In what follows, we explain the procedure employed to correlate this measured spectra with the concentration of the antibiotics.

#### 2.4.1. Reduction of Signal Dimensionality

The algorithm designed to detect the presence of antibiotic in raw milk assumed that the measured spectra are deterministic signals in noise. Such absorption spectrum can be described by the linear combination of known continuous-spectrum functions with unknown coefficients. Namely, the measured absorption spectra $r_{j}\left(\lambda \right)$ can be recast as $r_{j}\left(\lambda \right)\approx {\hat{r}}_{j}\left(\lambda \right)=\sum_{n=1}^{N}\alpha_{j,n}P_{n}\left(\lambda \right)$, where the subscript *j* stands the *j*-th antibiotic to be detected (i.e., j=1,2,3,4), $P_{n}\left(\lambda \right)$ are the known continuous-spectrum expansion functions and $\alpha_{j,n}$ are the unknown expansion coefficients that must be determined. For the purpose of this work, we use the Legendre functions of the first type as the continuous-spectrum expansion functions for all the measured spectra. By projecting each measured spectrum $r_{j}\left(\lambda \right)$, with j=1,2,…, onto a set of the first *N* expansion functions, namely ${\left\{P_{n}\left(\lambda \right)\right\}}_{n=1}^{N}$, we obtain the expansion coefficients by $\alpha_{j,n}=<r_{j}\left(\lambda \right),P_{n}\left(\lambda \right)>=\int_{\lambda_{1}}^{\lambda_{2}}r_{j}\left(\lambda \right)P_{n}\left(\lambda \right)\;d\lambda$, where $\lambda_{1}$ and $\lambda_{2}$ are the minimum and maximum wavelengths captured by VIS-NIR spectrometer. By using the approximation ${\hat{r}}_{j}\left(\lambda \right)$ as stated with this expansion coefficients we assure that ${\hat{r}}_{j}$ is the optimal approximation of $r_{j}$ in the mean-square sense.

**Figure 4:**
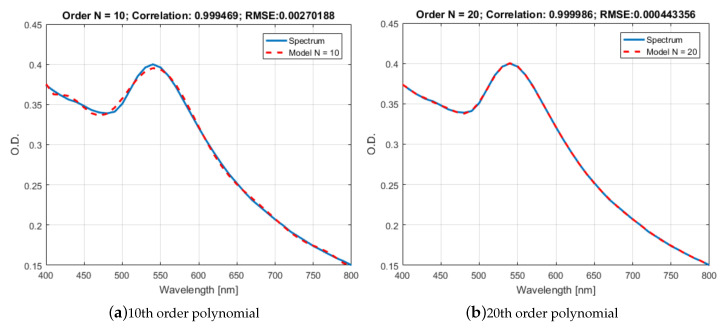
Adjustment of the measured absorption spectrum $r_{j}\left(\lambda \right)$ by means of Legendre functions of the first type. As the number of basis functions is increased, the better the fit of the approximation ${\hat{r}}_{j}\left(\lambda \right)$. The approximation is shown with (**a**) ten basis functions and (**b**) twenty basis functions.

In Figure 4, we show how the number of basis functions, used to synthesize $r_{j}$ improves the fit of the curves. The blue line represents one measured absorption spectrum of Kanamycin $r_{j}$ and the red line is the corresponding approximation ${\hat{r}}_{j}$ as the number of basis functions is changed from N=1 (see Figure 4a), up to N=20 (see Figure 4d).

**Figure 5:**
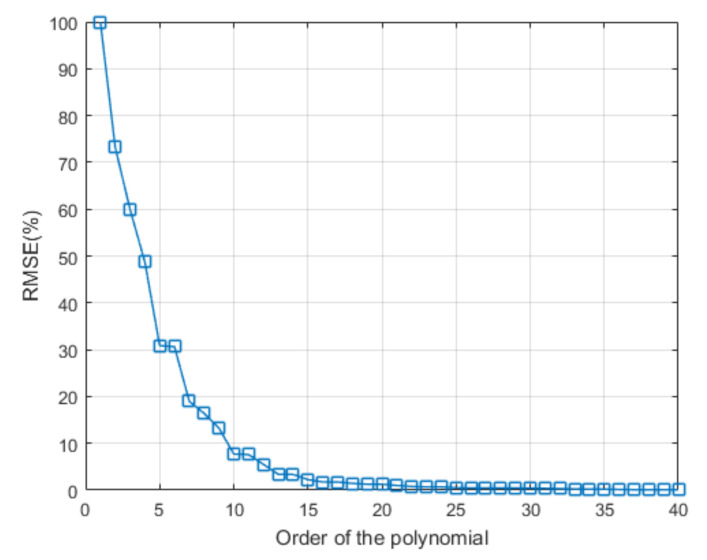
Root-mean-square error (RMSE) of the fit between the measured absorption spectrum $r_{j}\left(\lambda \right)$ and the approximation made with the Legendre functions ${\hat{r}}_{j}\left(\lambda \right)$ for Kanamycin. We require N=20 basis functions to ensure an RMSE below 1%.

Using the root-mean-square error between $r_{j}$ and ${\hat{r}}_{j}$ as a metric of the fit, we have determined that N=20 Legendre functions are required to guarantee a RMSE below 1% across all the possible concentrations of all the antibiotics used in this work. To illustrate this, in Figure 5 we depict the effect on the RMSE of adding more basis functions in approximate a family of measured spectra for Kanamycin.

We are now able to consider that N=20 basis functions are required to optimally fit all the measured spectra for all the used antibiotics with a maximum root-mean-square error (RMSE) of 1%. These functions are the basis for the space of absorption spectra. Then, for each measured absorption spectrum, the best fit is made using the first twenty Legendre functions and the coefficients are considered as the representation of the spectrum signal in this new space. Then, the *j*-th absorption spectrum $r_{j}\left(\lambda \right)$ within the set of available data is simply represented by its corresponding expansion coefficients $\alpha_{j}={\left[\alpha_{j,1}\;\;\alpha_{j,2}\;\;\ldots \;\;\alpha_{j,20}\right]}^{T}$. This involves a significant dimensional reduction at the cost of prepossessing each acquired spectra. Likewise, the curves will no longer be represented by high-resolution spectra but rather by only 20 expansion coefficients (adding 20 ms to the processing time). What is postulated at this time is that the correlation between the measured spectrum and the concentration of each antibiotic can be carried out successfully on the expansion coefficients.

#### 2.4.2. Design of the Antibiotic Detection Algorithm

**Figure 6:**
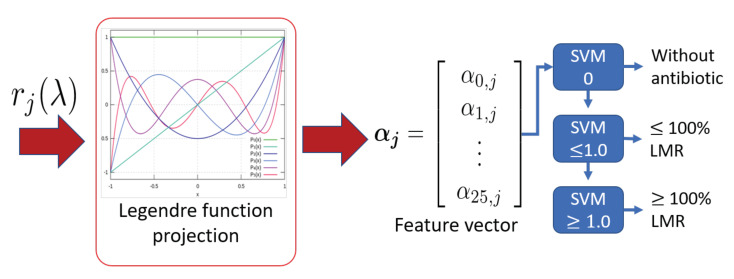
The spectrum measured from a sample, $r_{j}$, with unknown antibiotic presence and concentration level enters the algorithm. The support-vector machine (SVM) scheme determines whether an antibiotic is present or not. If the antibiotic is present in the sample, the tree is cascaded until one of the SVM is certain, meaning it has detected the concentration level.

For the purpose of this work, we employed support-vector machine (SVM) classifiers to determine the presence and the level of antibiotics concentration for a given spectrum. According to the Chilean regulation, we defined three classifiers: one to determine whether the sample has antibiotic or not (SVM 0), one to determine if the sample has antibiotic with a concentration below the MRL (SVM ≤ 1.0) and another to determine whether the sample has a concentration of antibiotic above the MRL (SVM ≥ 1.0). In Figure 6, one can see the classification scheme. The measured spectrum with unknown antibiotic concentration $r_{j}$ enters the algorithm from which the expansion coefficients $\alpha_{j}$ are computed. These coefficients are used to infer the concentration among the aforementioned classes: the sample has no antibiotic, the sample has antibiotic with concentration below or above the MRL.

The concentration assigned to each spectrum corresponds to the majority determined through the output of each of the classifiers. This is known in the literature as a “one-against-all” decision tree with a majority decision rule “winner-takes-all”.

#### 2.4.3. Description of the Dataset Used in This Work

In order to train the algorithms that determine the presence and concentration level of the different antibiotics, we utilized the absorption spectra of clarified milk. The NBSs for each antibiotic were incubated in clarified milk contaminated with different concentrations of antibiotics, namely 0 ppb of antibiotic (milk without antibiotic), 25% of the MRL, 50% of the MRL, the actual MRL of the antibiotic, 150% the MRL and 200% the MRL. It is understood that if the concentration of an antibiotic is higher than the MRL, then the raw milk does not comply the legal regulations.

For each of the aforementioned concentrations, we performed several experiments and measured the corresponding absorption spectra. There are 60 samples per antibiotic that contain 5 levels of concentration. Each of these experiments required the full process of NBS construction, contamination of the raw milk with each antibiotic at the given concentration, clarification of the contaminated raw milk, interference reduction and the acquisition of the absorbance spectra by the built-in instrument.

**Figure 7:**
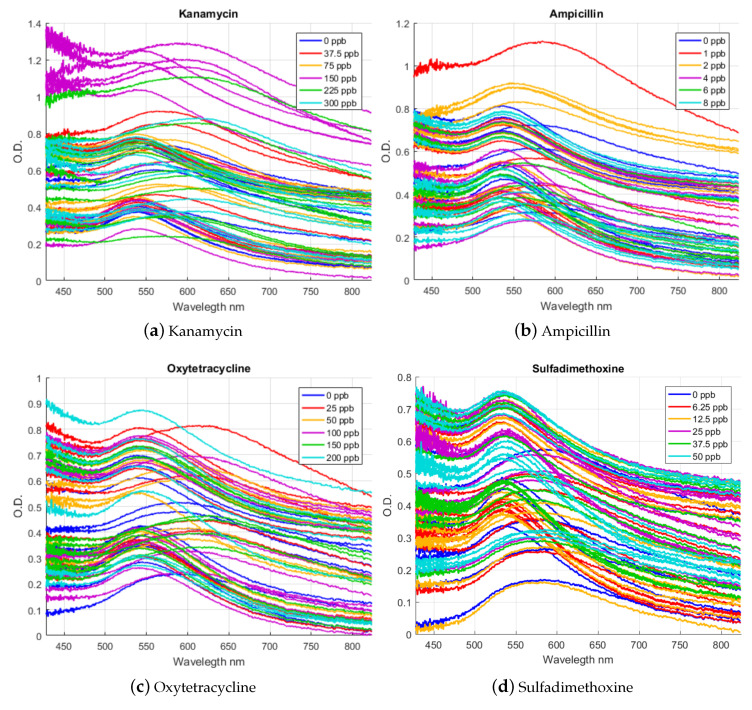
Measured absorption spectra for the four antibiotics used in this work. The available repetitions for each antibiotic and each of the concentrations are shown. Although the curves show separability, it is not possible to estimate concentrations by visual inspection.

In Figure 7 we show the absorption spectra measured for the four antibiotics used in this work. It can be noted that it is not possible to simply discriminate color differences by visual inspection, especially due to the observed variations in the measured spectrum for each repetition. To illustrate this, the measured spectrum for Kanamycin is shown in Figure 7a. The purple curves depict the measured spectrum when the concentration of Kanamycin is the actual MRL (150 ppb). It can be seen that the spectrum for each repetition differs significantly from each other, despite the concentration of antibiotics is the same. Moreover, in some cases the spectra overlap the repetitions of other concentrations of the antibiotic.

## 3. Results and Discussion

From the present results, considering a classification with a 100% success rate and that the NBSs were developed specifically to interact with each individual antibiotic, it is determined that the specificity of the NBS is 100%, by design, for all antibiotics. The specificity of the algorithm for estimating different concentrations was 100% for Kanamycin, 99.12% for Ampicillin, 91.67% for Oxytetracycline and 99.17% for Sulfadimethoxine. For Kanamycin, the sensitivity reached was 100%. For Ampicillin, the maximum sensitivity reached was 95.65%, with an average of 88.83%. For Oxytetracycline, the maximum sensitivity reached was 91.67%, with an average of 88.33%. Finally, for Sulfadimethoxine, the maximum sensitivity obtained was 95.83%, with an average of 91.48%. The linear range of all classifiers is 0 times MRLs up to 2 times the MRL. For all antibiotics, the detection limit was 0.25 times the MRL, corresponding to 37.5 ppb for Kanamycin, 1 ppb for Ampicillin, 25 ppb for Oxytetracycline and 6.25 ppb for Sulfadimethoxine, see Table 1. Note that the best results achieved are for Kanamycin since its optical response is more disaggregated into important parts of the measured spectral band than for the other antibiotics optical response, see Figure 7.

The data were analyzed through the standard methodology in order to compare the results (see a detailed description in [30]). The traditional metrics chosen were the absorbance ratios $A_{520}$/$A_{620}$, $A_{520}/A_{720}$ and the minimum absorbance over the maximum $A_{min}/A_{max}$. The detection limit can be obtained from the calibration curves. For this database, the following results were obtained: (i) for Kanamycin the best result was obtained for the $A_{520}/A_{620}$ ratio and its detection limit was 170.93 ppb; (ii) for Ampicillin the best result was also for the $A_{520}/A_{620}$ ratio and its detection limit was 2.977 ppb; (iii) for Oxytetracycline, the best result was also for the $A_{520}/A_{620}$ ratio and its detection limit was 232.95 ppb, (iv) for Sulfadimethoxine the best result was for the $A_{min}/A_{max}$ ratio and its detection limit was 44.66 ppb.

Finally, these results offer insight into the capability of the technology to provide confirmatory analysis for screening tests that are routinely applied in farms/diaries for rapid detection of antibiotic residues in raw milk (e.g., Delvotest${}^{®}$ and Charm${}^{®}$ kits). These kits allow for direct measurements on raw milk samples (often avoiding pre-processing), but they are less specific (detect groups/families of antibiotics) and provide semi-quantitative results. For example, Charm${}^{®}$ Rapid One Step Assay (ROSA) technology is extensively applied by the dairy industry, which meets the 100 ppb EU MRL requirement for Tetracycline drugs, a level which most countries have adopted in their milk regulations. Herein, we have proved for Oxytretracycline a detection limit of 0.25 times the MRL (i.e., 25 ppb), outperforming screening tests. Despite the process requiring pre-analytical treatment of the raw milk (similar to HPLC methods), the analysis is still fast (30 min) and sensor technology holds promise for portable ”on-farm” applications.

## 4. Conclusions

This article addressed the need to develop portable technology for on-site and sensitive detection of antibiotic residues in cow milk, before reaching the food chain. Accordingly, we have developed and investigated a prototype of an automatic electro-opto-mechanic system with the capability of measuring the optical absorption response of antibiotic-specific NBS in multiple samples of clarified milk. The prototype was successfully tested for the detection of Kanamycin, Ampicillin, Oxytetracycline and Sulfadimethoxine. We have developed effective support-vector machine (SVM) classifiers to determine the presence and concentration levels for the antibiotics, outperforming in sensitivity the typical metrics based on the absorbance ratio. The SVM-based classification algorithm allowed to reach a detection limit of 0.25 times the MRL, with a linear range from 0 to 2 times the MRL for each antibiotic.

Overall, this technology offers a combination of portability and sensitivity that supports further development in the field of confirmatory analysis for screening tests applied in farms and diaries to detect antibiotic residues in raw milk.

## Figures and Tables

**Table 1:** Accuracy rate, cross-validation results SVM (one-against-all) for 20 expansion coefficients. The average rate obtained for cross validation is shown, repeating the training with all combinations of the different sets.

Antibiotic	n	Average Rate	0 MLR	1 MLR	2 MLR
Kanamycin	1	100.00%	87.75%	100.00%	75.00%
	2	100.00%	87.75%	100.00%	100.00%
	3	100.00%	87.75%	100.00%	100.00%
Ampicillin	1	87.50%	83.33%	83.33%	75.00%
	2	83.33%	83.33%	83.33%	87.50%
	3	95.65%	86.96%	83.33%	75.00%
Oxytetracycline	1	90.00%	95.83%	100.00%	100.00%
	2	83.33%	80.00%	67.67%	100.00%
	3	91.67%	83.33%	60.00%	100.00%
Sulfadimethoxine	1	91.67%	91.67%	71.67%	62.50%
	2	86.96%	86.96%	66.67%	75.00%
	3	95.83%	87.50%	100.00%	100.00%

## Abbreviations

The following abbreviations are used in this manuscript:

AuNP	Gold nanoparticles
HPLC	High-performance liquid chromatography
ISP	Instituto de Salud Pública
NBS	Nanobiosensor
MRL	Maximum Residual Limit
RMSE	Root-mean-square error
SAG	Servicio Agrícola y Ganadero, Chile
SPR	Surface Plasmon Resonance
SVM	Support-vector machine
